# Supplementary material for: Generation of two multipotent mesenchymal progenitor cell lines capable of osteogenic, mature osteocyte, adipogenic, and chondrogenic differentiation
Source: Sci Rep. 2021 Nov 19;11:22593. doi: 10.1038/s41598-021-02060-1 (PMC8605002; doi:10.1038/s41598-021-02060-1)
Supplement: Supplementary file 1 — Supplementary Figures. [file 41598_2021_2060_MOESM1_ESM.pdf]

Generation of two Multipotent Mesenchymal Progenitor Cell Lines Capable of Osteogenic, Mature Osteocyte, Adipogenic, and Chondrogenic Differentiation

[1]Matthew Prideaux[#], [1,2]Christian S. Wright[#], [1,3]Megan L. Noonan, [2]Xin Yi, [1,3]Erica L. Clinkenbeard, [4]Elsa Mevel, [3]Jonathan A. Wheeler, [5]Sharon Byers, [5]Asiri R. Wijenayaka, [6,7]Stan Gronthos, [1,4]Uma Sankar, [1,3,5]Kenneth E. White, [5]Gerald J. Atkins*, [1,2,4]William R. Thompson*✉

[1]Indiana Center for Musculoskeletal Health, Indiana University, Indianapolis, IN 46202
[2]Department of Physical Therapy, School of Health and Human Sciences, Indiana University, Indianapolis, IN 46202
[3]Department of Medical and Molecular Genetics, Indiana University, Indianapolis, IN 46202
[4]Department of Anatomy, Cell Biology, & Physiology Indiana University, Indianapolis, IN 46202
[5]Centre for Orthopaedic & Trauma Research, Faculty of Health and Medical Sciences, University of Adelaide, SA, Australia, 5005
[6]Mesenchymal Stem Cell Laboratory, Faculty of Health and Medical Sciences, University of Adelaide, SA, Australia, 5005
[7]Precision Medicine Theme, South Australian Health and Medical Research Institute, Adelaide, SA, Australia

[#]Authors provided equal contribution to the work.
*Equal senior authors

✉Corresponding Author: William R. Thompson, DPT, PhD
Email: wrthomps@uab.edu
Ph.: (205) 975-2788

E-mail Addresses: mprideau@iu.edu; wrighch@iu.edu; mlnoonan@iu.edu; xinyi@iupui.edu; eclinken@iu.edu; elsa.mevel@hotmail.fr; jonwheel@iu.edu; sharon.byers@adelaide.edu.au; asiri.wijenayaka@adelaide.edu.au; stan.gronthos@adelaide.edu.au; usankar@iupui.edu; kenewhit@iu.edu; gerald.atkins@adelaide.edu.au; wrthomps@uab.edu

Supplementary Figures

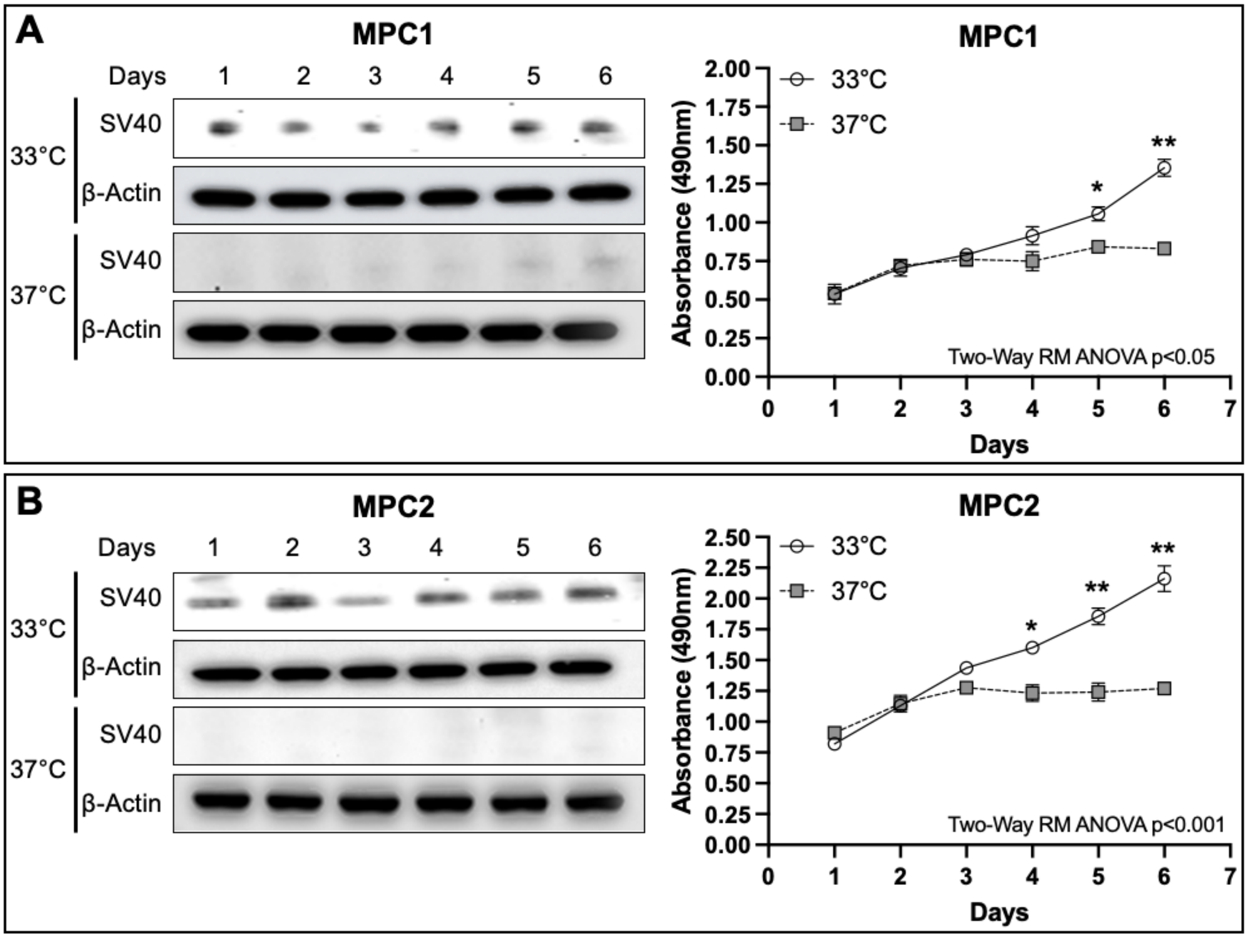

Figure S1: Growth Characterization of MPC cells. MPC cells were cultured in growth media (GM) for 1-7 days at 33°C and 37°C. Western blots were performed from MPC1 (A) and MPC2 (B) cell lysates. Blots were probed for SV40 and β-actin as a loading control. MTT assays were performed to assess cell viability in MPC1 (A) and MPC2 (B) cells grown at either 33°C or 37°C. Each sample was tested in triplicate (n=3; mean +standard error). *p<0.05, **p<0.01, and ***p<0.001 two-way repeated measures ANOVA with multiple comparisons denoted for individual timepoints.

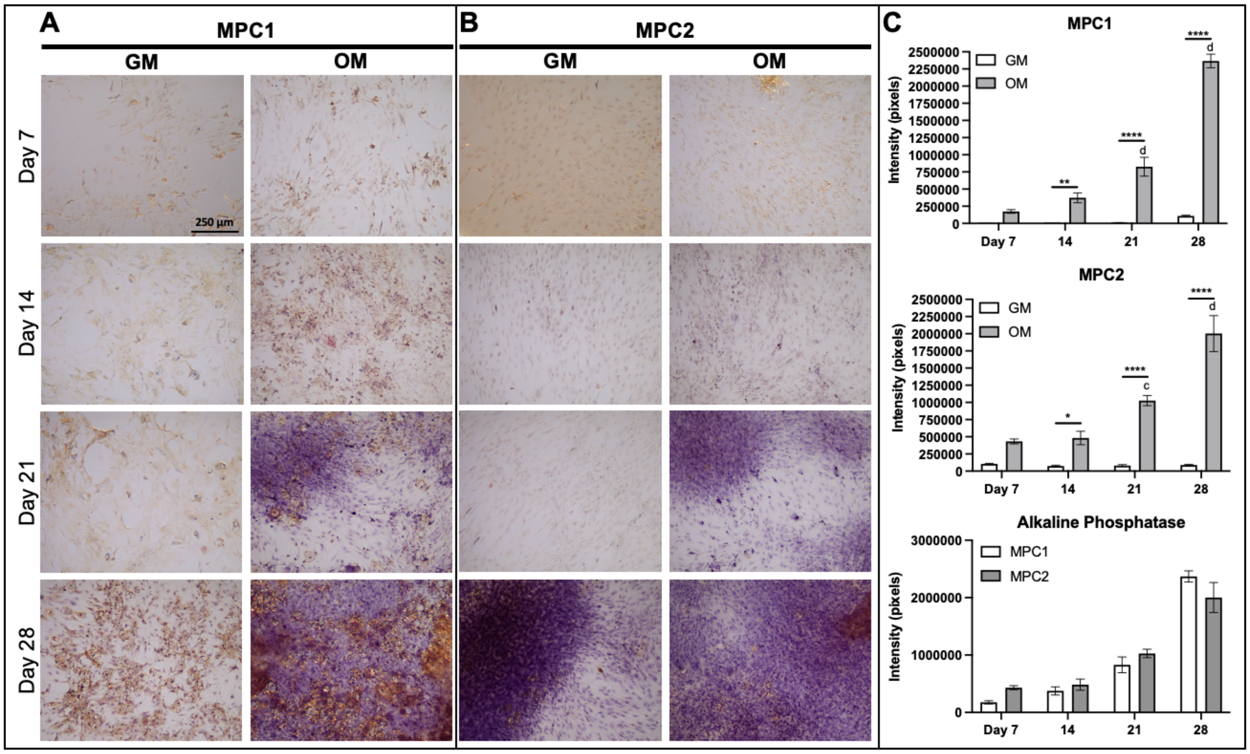

Figure S2: Osteogenic differentiation and alkaline phosphatase staining of MPC cells. MPC cells were cultured in growth media (GM) or osteogenic media (OM) and stained for alkaline phosphatase. (A) In MPC1 cells Alkphos staining began to appear in cultures grown in osteogenic media at day 14, with large differences at days 21 and 28 compared to growth media. (B) MPC2 cells displayed strong staining at day 21 in osteogenic media. After 28 days in culture MPC2 cells grown in growth media also had strong Alkphos staining. (C) Staining was quantified using ImageJ. Each cell line was tested in triplicate. Images were captured using a 10X magnification lens. (n=3; mean + standard error). *p<0.05, **p<0.01, ***p<0.001, and ****p<0.0001 between GM and OM at the time point designated.

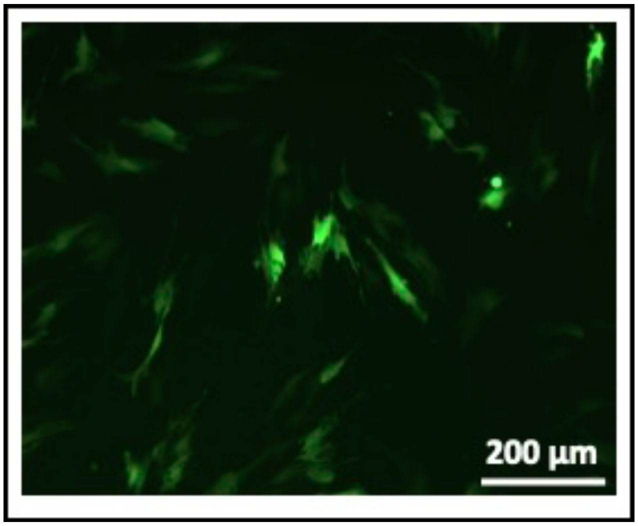

Figure S3: Transfection with GFP. To establish the ability of MPC cells to be transfected, MPC2 cells were cultured in growth media (GM) at 33°C. A vector containing eGFP was transfected into the cells using Fugene-6 HD. Images were captured using a fluorescent microscope (Leica) 24 h after transfection. (10X; bar = 200 µm)

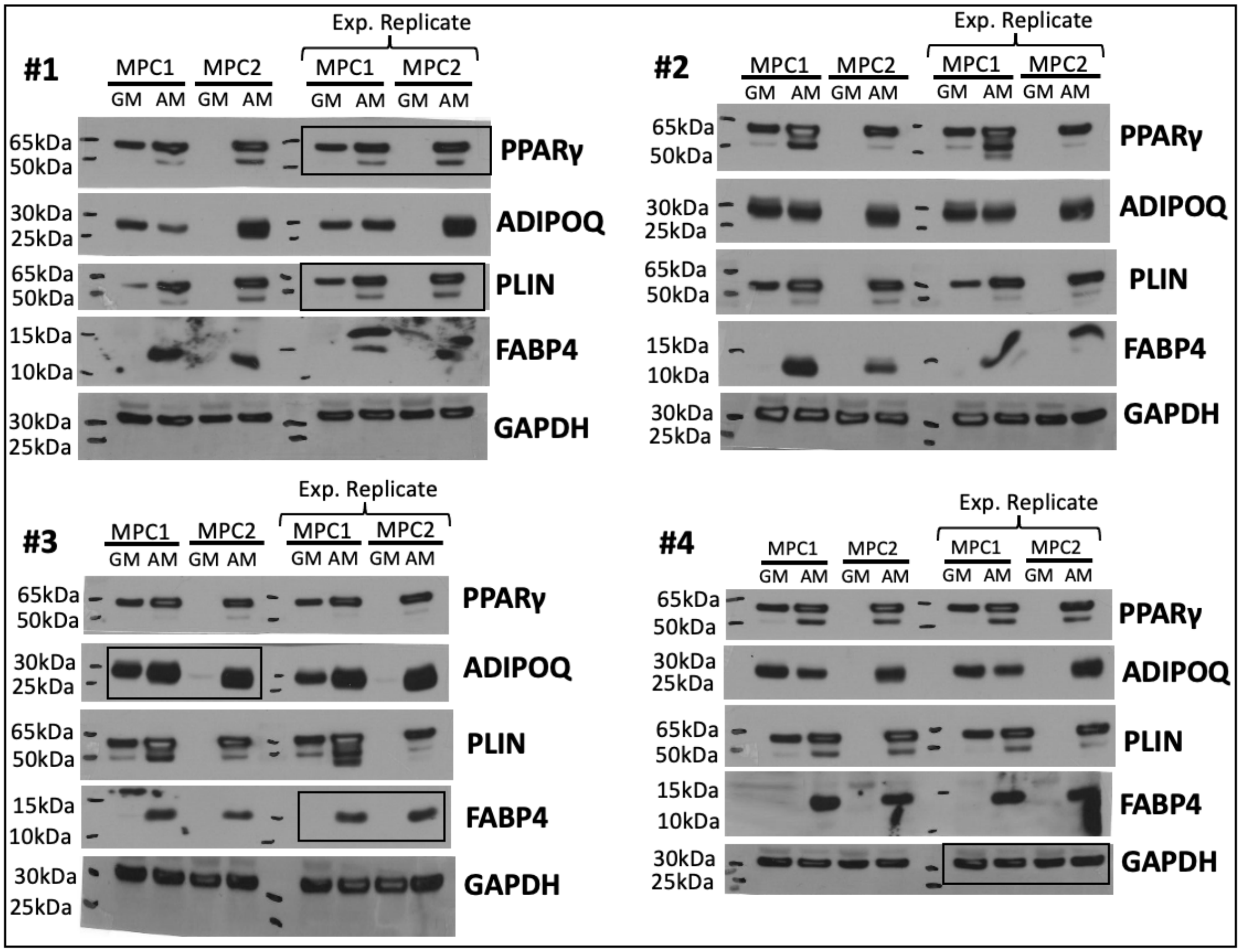

Figure S4: Original images of Western blots for adipogenic markers. Western blots used for densitometry quantification for MPC1 and MPC2 cell adipogenic differentiation assays. Four biological replicates were included, and each biological replicate was run twice on the blot, as indicated as an experimental replicated. Blot images used in the primary figure are outlined in black.

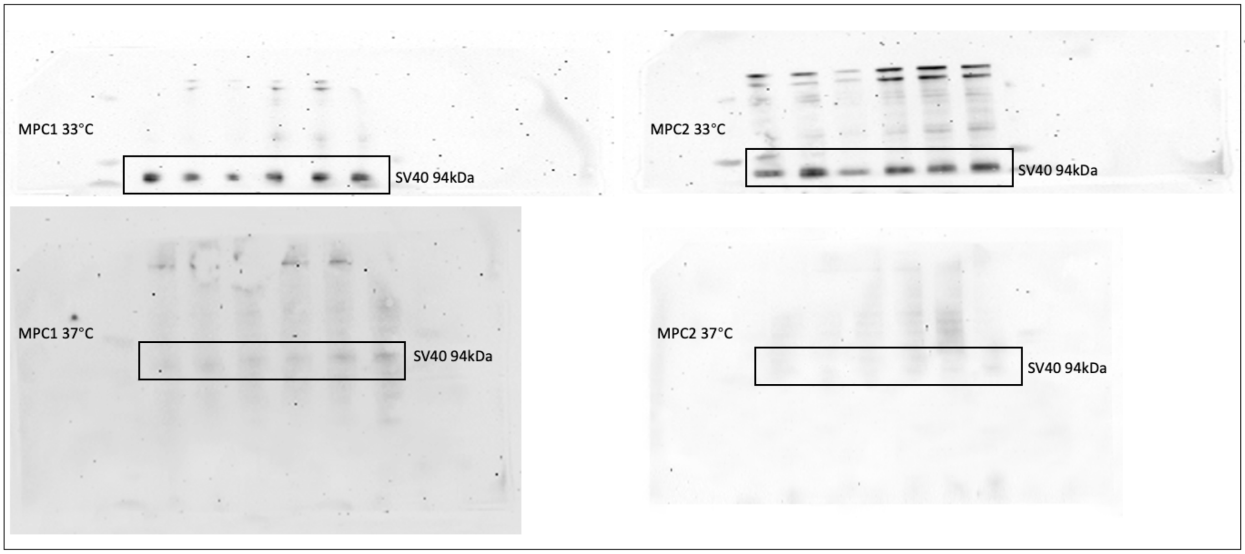

Figure S5: Full size un-cropped images of blots for SV40.

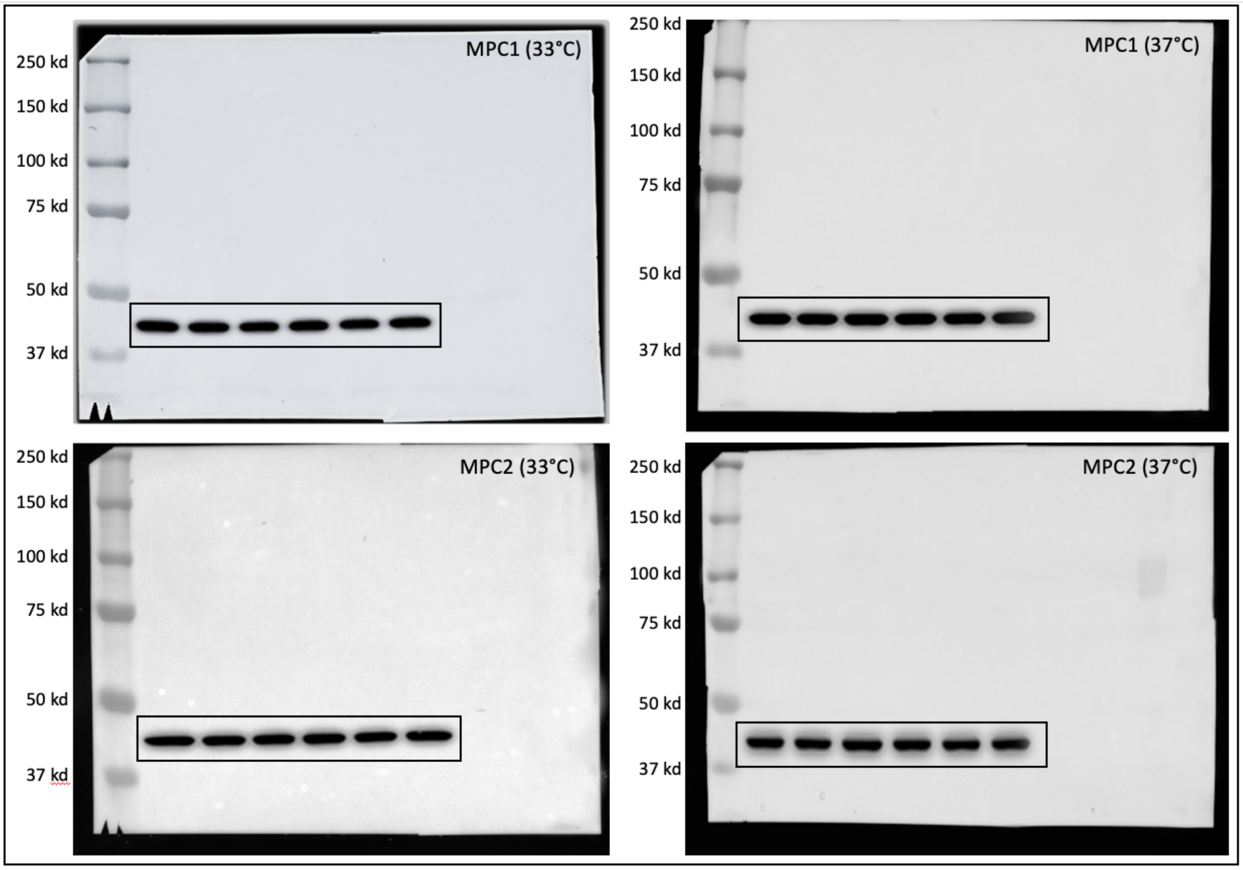

Figure S6: Full size un-cropped images of blots for β-actin.
